# Paternal alcohol exposure and dental-facial anomalies in offspring

**DOI:** 10.1172/JCI173607

**Published:** 2023-10-02

**Authors:** Divya Vinayachandran, Saravana Karthikeyan Balasubramanian

**Affiliations:** 1 Department of Oral Medicine and Radiology, SRM Kattankulathur Dental College and Hospital, Potheri, SRM Institute of Science and Technology, Tamil Nadu, India.; 2 Department of Conservative Dentistry and Endodontics, SRM Dental College, Ramapuram, SRM Institute of Science and Technology, Tamil Nadu, India.

**Keywords:** Development, Genetics, Addiction, Embryonic development

**To the Editor:** We read with great interest the recent article by Thomas et al. (1), wherein the authors shed light on an unexplored research hypothesis, highlighting the significant link between paternal alcohol exposure and fetal alcohol syndrome (FAS). Considering the seriousness with which alcohol use by women during pregnancy has been viewed, it is noteworthy that there is no previous research evidence on the role of paternal alcohol exposure in the development of craniofacial abnormalities and related growth deficiencies.

Using a multiplex mouse model study, Thomas et al. (1) showed observations, including alcohol-induced changes that predominated in the lower regions of the face, including the maxilla, mandible, and ear positioning, with a shift of midline features to the right, which is in accordance with previous literature (2). Craniofacial dysmorphology assessment included demarcation of 16 left/right profile and 18 front landmarks, of which those relating to the oral cavity (dentofacial region) included the bottom/front of the mandible, philtrum, and corner of the mouth. While we applaud the authors for their commendable research, we suggest that an extensive study of this nature could have also included assessment of the presence of cleft lip/palate, high arched palate, and other tooth developmental anomalies, such as variations in size (small/asymmetrical teeth), enamel hypoplasia, and alterations in alignment, which have all been reported previously in FAS (2, 3).

Evidence of genetic/environment factors in the etiology of clefts have been proposed, with ethanol acting as a competitive inhibitor of NAD^+^-dependent alcohol dehydrogenases (EC.1.1.1.1 ADH) that catalyzed retinal oxidation in embryonic tissues (4). This further leads to disruptions in the distribution of retinoic acid, a carboxylic acid metabolite of retinol (vitamin A) that plays a crucial role in specifying spatial patterns in embryogenesis (4). It has also been suggested that prenatal alcohol consumption exclusively affects the tooth germ early, before it is calcified (5). Tooth calcification occurs only after birth, and the effect of alcohol on the soft tissue stage of odontogenesis is primarily responsible for the fluctuating odontometric asymmetry, as seen in FAS.

Because earlier evidence indicates that congenital dentofacial anomalies have been found in cases of maternal alcohol exposure, it is imperative to conduct rigorous future investigations to assess whether paternal alcohol exposure also results in a similar pattern.

## Version 1 10/02/2023

Electronic publication

## References

[1] Thomas KN (2023). Preconception paternal ethanol exposures induce alcohol-related craniofacial growth deficiencies in fetal offspring. J Clin Invest.

[2] Klingenberg C (2010). Prenatal alcohol exposure alters the patterns of facial asymmetry. Alcohol.

[3] Clarren SK, Smith DW (1978). Incidence of fetal alcohol syndrome and prevalence of alcohol-related neurodevelopmental disorder. Teratology.

[4] Chase JR (2009). Contribution of NADH increases to ethanol’s inhibition of retinol oxidation by human ADH isoforms. Alcohol Clin Exp Res.

[5] Kieser JA (1992). Fluctuating odontometric asymmetry and maternal alcohol consumption. Ann Hum Biol.

